# Microbiome-Metabolome Responses in Ruminal Content and Feces of Lactating Dairy Cows With N-Carbamylglutamate Supplementation Under Heat Stress

**DOI:** 10.3389/fvets.2022.902001

**Published:** 2022-06-23

**Authors:** Yan Li, Ning Ma, Liyuan Ren, Meimei Wang, Linqi Hu, Yizhao Shen, Yufeng Cao, Qiufeng Li, Jianguo Li, Yanxia Gao

**Affiliations:** ^1^College of Veterinary Medicine, Veterinary Biological Technology Innovation Center of Hebei Province, Hebei Agricultural University, Baoding, China; ^2^College of Animal Science and Technology, Hebei Agricultural University, Baoding, China; ^3^Hebei Technology Innovation Center of Cattle and Sheep Embryo, Baoding, China; ^4^Hebei Research Institute of Dairy Industry Technology, Shijiazhuang, China

**Keywords:** cow, metabonomics, microbiota, N-carbamylglutamate, rumen

## Abstract

The objective of the present study was to investigate the effects of N-carbamylglutamate (NCG) supplementation on metabolic profile and microbiota in ruminal content and feces of lactating dairy cows under heat stress (HS). Forty-eight lactating Holstein cows (154 ± 13.6 days in milk) were assigned randomly to four treatments (*n* = 12), to receive 0, 15, 20, or 25 g/day of commercial NCG (proportion: 97.7%) for the period of 60 days. The recorded ambient temperature–humidity index (THI) suggested that the cows were exposed to HS for almost the entire experimental period (average THI: 80.6). Samples of ruminal content and feces were collected at the end of the trial (day 60) to determine the biological effects of NCG supplementation on metabolome and microbiota using mass spectrometry-based metabolomics and 16S rRNA gene sequencing techniques, respectively. Results showed that NCG supplementation enhanced the levels of ruminal microbial protein, total volatile fatty acids (VFAs), and the molar proportion of propionate in the rumen, but lowered the ruminal pH, ammonia nitrogen (NH_3_-N), and the ratio of acetate to propionate. NCG at doses of 20 and 25 g/day reduced the community richness and diversity of ruminal microbiota with the decrease of Shannon and Simpson diversity. Compositions of ruminal and fecal microbiotas were altered by NCG, and the PICRUSt results revealed that metabolic pathways of the bacteria, such as amino acid metabolism, energy metabolism, and pyruvate metabolism, were enriched in NCG groups. Distinct changes in the metabolomic profile of ruminal fluid were observed between the control and NCG groups. Changes of 26 metabolites mainly involved in arginine metabolism, glutamate metabolism, and nitrogen metabolism were observed associated with NCG supplementation. These results provided new insights into the effects of NCG on metabolomic profile and microbiota in ruminal content and feces, and the optimal dose of NCG supplemented to dairy cows was 20 g/hd/day, which contributed to understanding the effects of NCG on HS in lactating dairy cows.

## Introduction

The gut microbiota plays a key role in nutrition acquisition, and gut microbiota improvement can increase the efficiency of ruminal fermentation and feed utilization in ruminant production (1). The structure and abundance of the gut microbiota are affected by many factors such as antibiotics, age, and dietary structure (2). It is well known that ruminal content and feces are important biological samples to investigate the changes in gut microbiota in dairy cows. A previous study found that the diversity and composition of fecal microbiota in dairy cows under summer heat stress (HS) were changed, and these changes were related to cows' health (3). Moreover, the studies found that the microbial community composition, bacterial diversity, and richness are different in feces and ruminal content (4). Gut microbiota also affects the host metabolism, immune response, and other physiological processes through the production of bioactive metabolites such as vitamins, amino acids, and volatile fatty acids (VFAs) (5). Recent studies have proved that microbial metabolites can be present in the plasma and other body fluids, and in this way, the gut microbiota can make interactions with the host (6, 7). Thus, the integrative analysis of gut microbiota and metabolite profiling can help us to improve animal production performance by increasing the understanding of interactions between the host and microbiota (8).

Dairy cows are sensitive to the high-temperature environment. With the aggravation of the greenhouse effect, HS causes more and more harm to dairy production, reproductive performance, and economic loss in the dairy industry (9, 10). HS can change the ruminal function and shift the composition and community structure of ruminal microbiota. Previous studies have found that ruminal pH and acetate concentration were decreased in cows under HS, whereas lactate concentration and energy-related bacteria such as *Streptococcus ruminobacter* and *Treponema* were increased (11). HS can also change the fecal microbial population, microbiota diversity, and composition, resulting in functional disorders in the immune system and lipid metabolism (3, 12).

Arginine is one of the essential amino acids for dairy cows. In addition to being a building block of protein synthesis, arginine is also used to convert into biological molecules such as nitric oxide, polyamines, and creatine, which play important roles in animal development and health. Dou et al. (13) reported that arginine could reduce the damage in intestinal epithelial cells of dairy cows caused by HS by lowering inflammation and oxidant stress. However, the short biological half time, ruminal degradation, and the competition in the transportation of alkaline amino acids limit the application of arginine as a feed additive in dairy farming (14–16). N-carbamylglutamate (NCG) can increase endogenous arginine synthesis through the activation of carbamyl phosphate synthetase-1 (17). Currently, a growing number of studies have indicated that NCG can be used as a promising feed additive in the livestock industry because NCG treatment can increase the growth rate, muscle protein synthesis, reproductive efficiency, and fetal development in mammals (18–20). However, as a feed additive enhancing arginine synthesis, the effects of NCG on HS in dairy cows remain unknown.

In dairy studies, NCG has been reported to improve production performance and milk quality (21). Our previous study found that NCG supplementation had no effects on dry matter intake and regulated plasma metabolomics profile in dairy cows under HS. Meanwhile, NCG increased milk yield, milk protein percentage, serum glucose concentration, and improved antioxidant capacity and immune function in dairy cows, which indicated the improving effect of NCG in HS (22). However, little information is known concerning the effects on metabolite profiles of ruminal fluid and gut microbiota associated with NCG treatment in dairy cows under HS. In conjunction with our previous study, it is necessary to further investigate the action mechanism of NCG on HS by ruminal fluid and gut microbiota.

16S rRNA gene sequencing technology has served as an important tool for the analysis of the structure and relative abundance of microbial communities. Metabolomics based on liquid chromatography mass spectrometry (LC-MS) is widely used to obtain metabolic information in biological systems (23). Notably, 16S rRNA gene sequencing coupled with metabolomics has been widely applied to investigate the effects of food type in dairy cows on the changes of microbiota and key metabolic pathways through the comprehensive analysis of biological samples such as milk, plasma, ruminal fluid, and urine (24–26). In this study, we applied LC-MS-based metabolomics to characterize ruminal metabolites and 16S rRNA gene sequencing technology to determine the changes in the ruminal and fecal microbial population in dairy cows treated by NCG under HS. The comprehensive analysis of the metabolites and microbiota offered important insights into metabolic processes and the microbial variations in the rumen and feces, which help to understand the mechanism of improved efficiency by NCG supplementation in dairy cows.

## Materials and Methods

### Animals, Diets, and Experiment Design

Forty-eight lactating Holstein cows (154 ± 13.6 days in milk, 1–3 parity and similar body condition score, BCS) from Hong Da dairy farm (Baoding, Hebei) were used in a randomized complete design and randomly assigned to four treatments (*n* = 12). The measurement of BCS was performed according to previous reports (27). In each treatment, 0 (control), 15 (low dose, LNCG), 20 (medium dose, MNCG), and 25 g/hd/day (high dose, HNCG) of NCG was precisely supplemented by mixing with total mixed rations (TMR) in the morning, respectively (20). The dairy cows were grouped to receive NCG supplementation treatment and free access to freshwater. Commercial NCG used in this study was purchased from Beijing Animore Sci. & Tech. Co., Ltd. (Beijing, China), the proportion of NCG was 97.7% and the rest were water and impurities. All cows were fed a standard TMR diet, the compositions and nutrient levels of TMR are listed in Supplementary Table S1. The experiment was conducted for 60 days. Ambient temperature and relative humidity of each day were measured to calculate the temperature-humidity index (THI) (28). With the use of a thermo-hygrometer probe (Model: AR837, XiMa Smart Sensor Co., Ltd., Hongkong, China), the temperature and relative humidity of cowsheds were measured in the morning (06:30), midday (14:00), and night (20:00), three times a day. As shown in Supplementary Figure S1, the THI was >72, indicating that the cows were under HS during the entire experiment.

### Sample Collection and Measurements

Five cows from each group were selected randomly for ruminal collection on day 60. Ruminal samples were collected using an oral stomach tube for 4 h after morning feeding, and the ruminal fluid samples were harvested by filtering ruminal contents with sterile four-layer cheesecloth (29). The oral stomach tube was inserted to a depth of 180 cm for ruminal fluid collection. In order to avoid ruminal fluid contamination from saliva or mucus in the mouth and esophagus, the first 150 ml of ruminal fluid was discarded, and then 150 ml of ruminal fluid was collected (29). The collected 150 ml ruminal contents were mixed evenly, divided into three 10 ml sterilization tubes, and quickly stored in liquid nitrogen. After being returned to the laboratory, they were stored at −80°C for ruminal microbiota analysis. The fecal samples were collected from the rectum using sterile gloves on day 60 (one pair of gloves per cow). The obtained fecal samples were divided into three 5 ml sterilized frozen tubes and put into liquid nitrogen immediately, and then the samples were stored in a refrigerator at −80°C for microbiota analysis.

The pH of the ruminal fluid was immediately measured using a digital pH meter (DENVER UB-7, Denver Instrument, Denver, United States). Two parts of the ruminal fluid samples (10 ml) were transferred to the refrigerator at −80°C within 2 h for metabolomic and VFAs analysis. In VFAs analysis, the ruminal fluid samples were acidified with 25% metaphosphoric acid, followed by standing for 30 min, and then centrifuged (10,000 *g*, 15 min). The supernatant was collected for VFA analysis using gas chromatography [7890A, Agilent Technologies, Chandler, United States, (30)]. An aliquot of ruminal fluid (10 ml) was acidified with 6 mol/L hydrochloric acids and stored at −20°C for the analysis of NH_3_-N concentrations (31). Ruminal microbial protein (MCP) was determined according to the previous study (32).

### DNA Extraction and Sequencing of Ruminal and Fecal Microbiota

Microbial DNA was extracted from ruminal contents using PowerFecal™ DNA Isolation kit (MO BIO Laboratories, Carlsbad, CA, United States), and the concentration and purity of DNA samples were determined by Nanodrop spectrophotometer (Thermo Scientific, Wilmington, NC, United States). 515f/806r primer set targeted the V4 region was used as PCR primer for 16S rDNA amplicon libraries. The PCR reactions were performed using Phusion High-fidelity PCR Master Mix (New England Biolabs LTD., Shanghai, China), and the amplified products were detected and purified by Qiagen Gel Extraction Kit (Qiagen, Hilden, Germany). Following amplification, sequencing libraries were constructed and then sequenced on an Illumina HiSeq 2500 platform at Novogene Technology Co., Ltd. (Tianjin, China) according to standard protocols.

### Bioinformatics Analysis for 16S rDNA Sequencing

Based on the unique barcode and primer sequence, the paired-end reads were assigned to the samples. Then, with the use of FLASH (Version 1.2.7), the paired-end reads were merged to obtain raw tags (33). For high-quality clean tags, raw tags were filtered by QIIME (Version 1.7.0) with the main parameters as follows: minimum quality score = 19, minimum/maximum length = 200/1,000, and error-correcting Golay 12 nucleotide barcodes (34). Tags were clustered to operational taxonomic units (OTUs) using UPARSE (Version 7.1) with a 97% similarity cutoff. Chimeras were filtered out using UCHIME (Version 4.2.40). The indices of Alpha diversity including Shannon, Chao1, Simpson, and ACE were calculated using MOTHUR (version v.1.30.1). Beta diversity was calculated with QIIME (Version 2.0) and displayed with R software (Version 2.15.3). Cluster analysis was performed by principal coordinate analysis (PCoA) to exhibit the differences of the samples using the WGCNA package, stat packages, and ggplot2 package in R software. Phylogenetic investigation of communities by reconstruction of unobserved states (PICRUSt) analysis was used to assess the metabolic changes of the microbiota in ruminal content and feces (35).

### Extraction of Ruminal Fluid Metabolites

Prior to analysis, the ruminal fluid samples were thawed on ice. Next, 200 μl of the sample was placed into a centrifuge tube, and 400 μl of methanol/water (4:1, v/v) was added. After vortex-mixing for 30 s, the mixture was incubated (1 h at −20°C) and centrifuged (14,000 *g*, 20 min at 4°C). And then, the supernatant of the ruminal fluid sample was further collected and dried. The obtained lyophilized powder was reconstituted (60% methanol, 100 μl), vortex-mixed (1 min), and centrifuged (14,000 *g*, 15 min at 4°C) for metabolomic analysis. Quality control (QC) samples were prepared by pooling equal volumes of supernatant from each sample and placed in the analysis sequence for system evaluation.

### Metabolomic Data Acquisition and Analysis

Metabolomic data acquisition was carried out by Vanquish ultra high-performance liquid chromatography (UHPLC) system coupled to a Q ExactiveTM HF-X mass spectrometer (Thermo Fisher, Waltham, MA, United States). Metabolomic data acquisition and analysis were performed according to previous studies (25). The detailed parameters of the UHPLC system, mass spectrometer, and data processing method are provided in Supplementary Material S1.

### Metabolite Identification and Pathway Analysis

With exacted molecular weight and MS/MS fragments, online databases such as HMDB (www.hmdb.ca/), METILN (metlin.scripps.edu), and Mass Bank (www.massbank.jp/) were used to identify the chemical structures of metabolites. For further biological interpretation, pathway analysis and biochemical reactions of the metabolites were conducted by MetaboAnalyst 4.0 software (www.metaboanalyst.ca/) and KEGG (www.kegg.jp/).

### Statistical Analysis

Data were analyzed by Proc Mixed of SAS 9.4 (SAS Institute Inc., Cary, NC) for a randomized complete design. In the MIXED model, NCG supplementation dose was a fixed effect and dairy cows were random variables. The linear, quadratic, and cubic NCG dose responses were determined by using specific pre-planned contrasts. The liner, quadratic, and cubic NCG dose responses were determined by using specific pre-planned contrasts. Data were reported as mean and standard error of mean (SEM). Significant differences were declared at *P* ≤ 0.05, and the tendency for a difference was declared at 0.05 < *P* ≤ 0.10.

## Results

### Effects of NCG on Ruminal Fermentation Index and Volatile Fatty Acids

Table 1 shows the effects of NCG supplementation on pH, NH_3_-N, MCP, and VFAs. The ruminal pH decreased linearly with the increase of NCG (*P* = 0.02). Compared with the control group, ruminal concentrations of NH_3_-N in the rumen in NCG-treated groups also decreased linearly (*P* < 0.01) with increasing NCG doses. The concentration of MCP cubically (*P* = 0.02) increased with NCG doses, where the medium dosage of NCG showed the best effect on MCP. There were quadratic (*P* = 0.01) and cubic (*P* < 0.01) effects of NCG levels on propionate and total VFAs, with the highest propionate and total VFAs at 20 g/hd/day NCG. The molar proportion of acetate tended to decrease linearly (*P* = 0.01). NCG dosage tended to have cubic effects on the molar proportion of butyrate (*P* = 0.01), and the ratio of acetate/propionate reduced quadratically with the increase of NCG (*P* = 0.01).

**Table 1:** Effects of NCG supplementation on ruminal fermentation patterns of lactating dairy cows under heat stress.

**Items**	**Groups**				**SEM**	**Probability**		
	**Control**	**LNCG**	**MNCG**	**HNCG**		**Linear**	**Quadratic**	**Cubic**
pH	6.6	6.3	6.2	6.2	0.11	0.02	0.38	0.67
NH_3_-N, mg/dl	13.1	11.8	10.4	11.2	0.42	<0.01	0.38	0.06
MCP, mg/ml	1.2	1.3	1.5	1.3	0.06	0.03	0.04	0.02
Total VFA, mmol/L	102.3	125.3	141.6	114.5	2.73	<0.01	<0.01	<0.01
Acetate, mol/100 mol	59.5	56.5	56.7	57.7	0.61	0.01	0.02	0.96
Propionate, mol/100 mol	27.1	31.4	33.1	30.9	0.51	0.01	0.01	0.02
Butyrate, mol/100 mol	13.4	12.1	10.2	11.4	0.41	0.01	0.31	0.01
Acetate/propionate	2.2	1.8	1.7	1.9	0.05	0.01	0.01	0.17

*NCG, N-carbamylglutamate; Control, without NCG supplementation; LNCG, NCG at 15 g/day per cow; MNCG, NCG at 20 g/day per cow; HNCG, NCG at 25 g/day per cow; MCP, microbial protein; SEM, standard error of mean*.

### Microbiota in Ruminal Content and Feces

**Figure 1:**
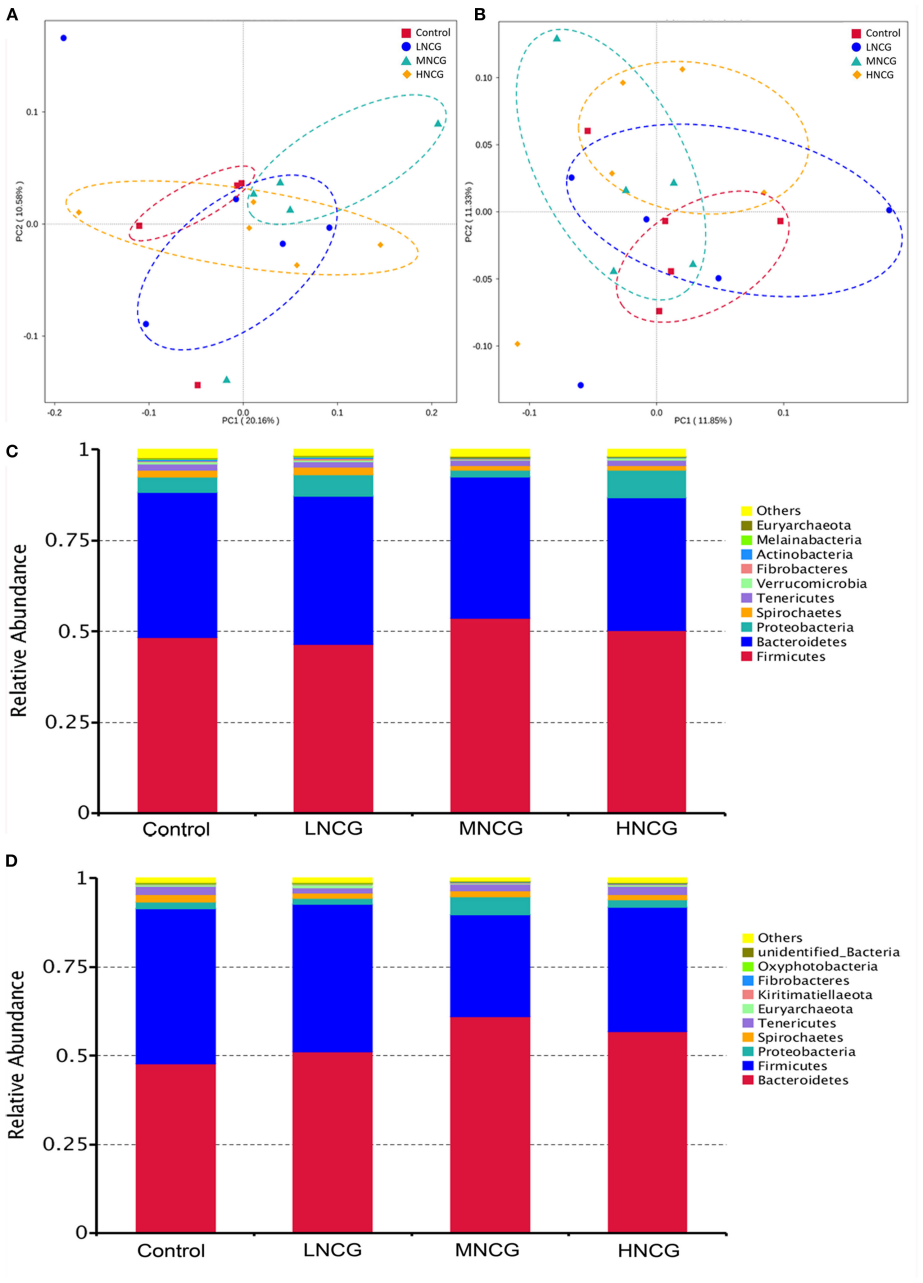
Effects of NCG supplementation on microbiota structure and composition in ruminal content and feces. NCG, N-carbamylglutamate; Control, without NCG supplementation; LNCG, NCG at 15 g/day per cow; MNCG, NCG at 20 g/day per cow; HNCG, NCG at 25 g/day per cow. **(A,B)** Principal coordinate analysis (PCoA) of bacterial community structures in ruminal content **(A)** and feces **(B)**. PCoA shows the difference in the bacterial communities among control and NCG groups. **(C,D)** The composition of the ruminal **(C)** and fecal **(D)** microbial community at the phylum level.

Effects of NCG on ruminal and fecal microbiota were evaluated by 16S RNA gene sequencing. A total of 19 ruminal contents and 20 fecal samples were used for sequencing. A summary of the sequencing data is provided in Supplementary Table S2. In ruminal and fecal samples, 2,159 and 2,272 OTUs were identified, respectively. Meanwhile, the results of rarefaction analysis indicated that the sequencing depth of the samples was sufficient to reflect the abundance and diversity of the microbiota (Supplementary Figure S2). Alpha diversity and beta diversity were used to evaluate the changes in microbial communities. Table 2 shows the summary of the Alpha diversity of the bacterial community. For ruminal content, no effect of NCG levels was observed on Simpson (*P* > 0.05), but Shannon (*P* = 0.01), Chao1 (*P* = 0.01), and ACE (*P* = 0.02) were reduced linearly with NCG dosage. In regard to the feces, the response of Shannon (*P* = 0.05) and Simpson (*P* = 0.03) to the increased dosage of NCG was cubic. Plots of PCoA revealed the clustering of ruminal and fecal samples in different groups, indicating the difference in the bacterial community structure (Figures 1A,B). At the phylum level, the results of OTU analysis of the ruminal contents are shown in Figure 1C. The relative abundance of ruminal microbiota at the phylum level was not affected by NCG treatment (Supplementary Table S3). In the feces, Bacteroidetes and Firmicutes were abundant in all groups (Figure 1D). The relative abundance of *Firmicutes* (*P* = 0.05) and *Proteobacteria* (*P* = 0.04) was reduced cubically with increased NCG (Supplementary Table S3). The *P*-value for quadratic contrast on *Tenericutes* was significant (*P* = 0.04), with the cows supplied NCG at 15 g/day having a lower abundance than cows supplied 20 or 25 g/hd/day of NCG. Linear discriminant analysis effect size (LEfSe) analysis was performed to identify the bacteria that were significantly different among the control and three NCG treatment groups. Supplementary Figure S3 displays the bacterial taxa represented in ruminal content and feces. In ruminal content, four taxa were overrepresented in the control, while five bacterial taxa were abundant in LNCG (Supplementary Figure S3A). No significant differences in the abundance of bacterial taxa in ruminal content were found between MNCG and HNCG groups. As to the feces, 22 bacterial taxa were detected in the LNCG and MNCG, and 19 distinct taxa in the control (Supplementary Figure S3B). Based on the relative abundance and statistical difference, seven genera in ruminal content and eight genera in feces were identified (Supplementary Table S4). Among them, *unidentified Prevotellaceae* in feces was increased cubically with increasing NCG (*P* = 0.03, Supplementary Table S4), while *Acetitomaculum, Marvinbryantia*, and *Unidentified Christensenellaceae* were reduced cubically (Supplementary Table S4). Meanwhile, the relative abundance *Mogibacterium* in ruminal content was reduced linearly with raising the dosage of NCG (*P* < 0.01).

**Table 2:** Summary of the Alpha diversity of the bacterial community in ruminal and fecal microbiota in different groups.

**Items**	**Groups**				**SEM**	**Probability**		
	**Control**	**LNCG**	**MNCG**	**HNCG**		**Linear**	**Quadratic**	**Cubic**
**Rumen content**
Shannon	8.70	8.12	8.09	7.88	0.192	0.01	0.78	0.68
Simpson	0.99	0.98	0.98	0.97	0.008	0.11	0.70	0.31
Chao1	2,332	2,158	2,018	2,056	74.3	0.01	0.71	0.35
ACE	2,328	2,178	2,028	2,073	76.2	0.02	0.79	0.32
**Feces**
Shannon	8.77	8.53	7.82	8.34	0.229	0.05	0.57	0.05
Simpson	0.99	0.99	0.97	0.98	0.007	0.16	0.68	0.03
Chao1	2,331	2,201	2,222	2,196	64.5	0.14	0.61	0.71
ACE	2,352	2,206	2,241	2,201	66.3	0.12	0.60	0.59

*NCG, N-carbamylglutamate; Control, without NCG supplementation; LNCG, NCG at 15 g/day per cow; MNCG, NCG at 20 g/day per cow; HNCG, NCG at 25 g/day per cow; SEM, standard error of mean*.

**Figure 2:**
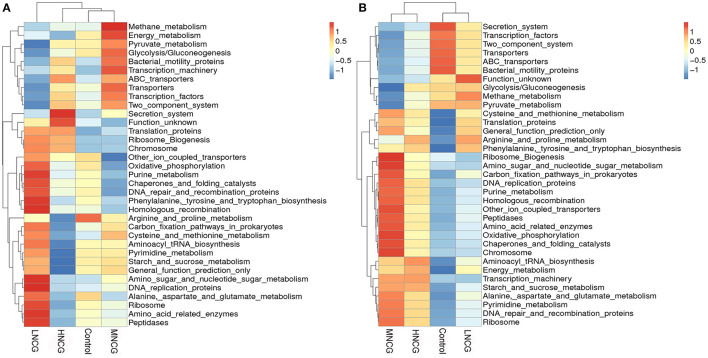
Heatmap of bacterial metabolic pathways obtained from PICRUSt analysis. NCG, N-carbamylglutamate; Control, without NCG supplementation; LNCG, NCG at 15 g/day per cow; MNCG, NCG at 20 g/day per cow; HNCG, NCG at 25 g/day per cow. PICRUSt analysis, Phylogenetic investigation of communities by reconstruction of unobserved states analysis. Microbiota functions in ruminal content **(A)** and feces **(B)** were analyzed by PICRUST. The ruminal and fecal microbiota function in NCG treatment groups were differed from those in the control that metabolism of energy, amino acid, pyruvate, glycolysis, and carbon fixation were enhanced in NCG groups.

The functional capacity of the microbiota in ruminal content and feces was inferred by the PICRUSt analysis. Heatmap of PICRUSt results (level 3) showed that the control group and NCG groups clustered differently, and differences in KEGG metabolic pathways were also observed in various groups (Figure 2). In the ruminal content, oxidative phosphorylation, purine metabolism, phenylalanine, tyrosine, and tryptophan biosynthesis, and alanine, aspartate, and glutamate metabolism were up-regulated in the LNCG compared with the control. Meanwhile, methane metabolism, energy metabolism, pyruvate metabolism, and glycolysis/gluconeogenesis were up-regulated in the MNCG but not in LNCG (Figure 2A). Interestingly, pyrimidine metabolism and arginine and proline metabolism were down-regulated in the HNCG. In feces, pathways of purine metabolism, oxidative phosphorylation, and pyrimidine metabolism were up-regulated in the MNCG, and the adverse effects were observed in the LNCG (Figure 2B).

### Ruminal Fluid Metabolic Profiling

**Figure 3:**
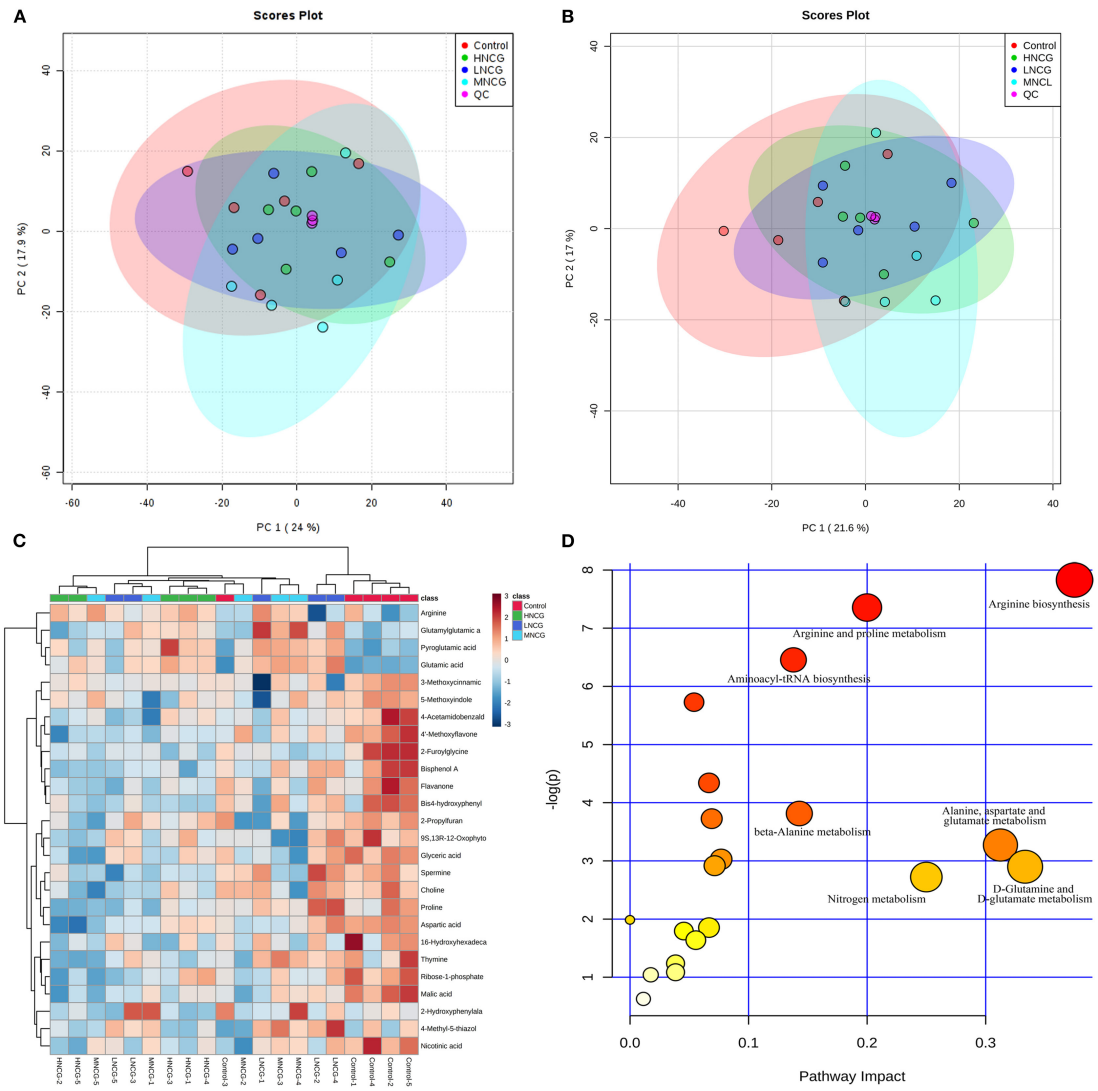
Effects of NCG supplementation on metabolomic profile in ruminal fluid. NCG, N-carbamylglutamate; Control, without NCG supplementation; LNCG, NCG at 15 g/day per cow; MNCG, NCG at 20 g/day per cow; HNCG, NCG at 25 g/day per cow. **(A,B)** PCA score plots of the ruminal fluid from the control and NCG groups analyzed by UPLC-Q-TOF/MS analysis in positive **(A)** and negative modes **(B)**. PCA, principal component analysis; QC, quality control samples. **(C)** Hierarchical clustering heatmap of the identified metabolites. The relative intensity of the metabolites was indicated by the color of the heatmap. **(D)** Pathway analysis of the metabolites obtained from ruminal fluid in response to NCG supplementation.

In order to validate the repeatability and stability of the method, pooled QC sample was applied in the ESI positive and negative analysis sequence, respectively. Principal component analysis (PCA) was first performed on all the samples in the study to find out the metabolic distinction. The distribution of the QC samples is shown in Figures 3A,B. Three QC samples were tightly clustered, which suggested that the method was robust with good suitability in the metabolomic experiment. Meanwhile, the control group and NCG-treated groups showed a slight but not significant separation trend in the PCA score plots in both ESI modes (Figures 3A,B).

The partial least squares discriminant analysis (PLS-DA) model was built to further identify the molecular features differentiated among the groups. Clear separation of the samples between control and NCG-treated groups could be observed in the PLS-DA score plots in both ESI positive and negative modes (Supplementary Figure S4), indicating there existed significant discrimination in ruminal fluid metabolic profile. Variables with VIP >1 and *P*-value <0.05 were selected as potential metabolites. As a result, 26 metabolites were identified as potential biomarkers related to NCG treatment (Supplementary Table S5). Compared with the control, 20 metabolites were reduced significantly, and 5 metabolites showed a remarkable increase in concentration in the NCG-treated groups. According to the metabolite abundance, the hierarchical clustering analysis was carried out to display the relationships and differences of the ruminal liquid metabolites. As shown in Figure 3C, metabolites in the same metabolic pathways or similar abundance pattern were clustered together. Heatmap also revealed that these metabolites significantly differed between the control and NCG-treated groups in ruminal liquid.

In order to visualize the metabolic pathways in response to NCG treatment, pathways analysis of the potential metabolites was performed by MetaboAnalyst. In this study, *Bos taurus* (cow) pathway library was selected and the pathway impact-value threshold was set to 0.10. Figure 3D showed that the potential metabolites were mainly involved in seven metabolic pathways, including arginine biosynthesis, arginine, and proline metabolism, aminoacyl-tRNA biosynthesis, nitrogen metabolism, D-glutamine, and D-glutamate metabolism, beta-alanine metabolism, and alanine, aspartate and glutamate metabolism.

### Correlation Among Metabolites, Microbiota, and Fermentation Parameters

**Figure 4:**
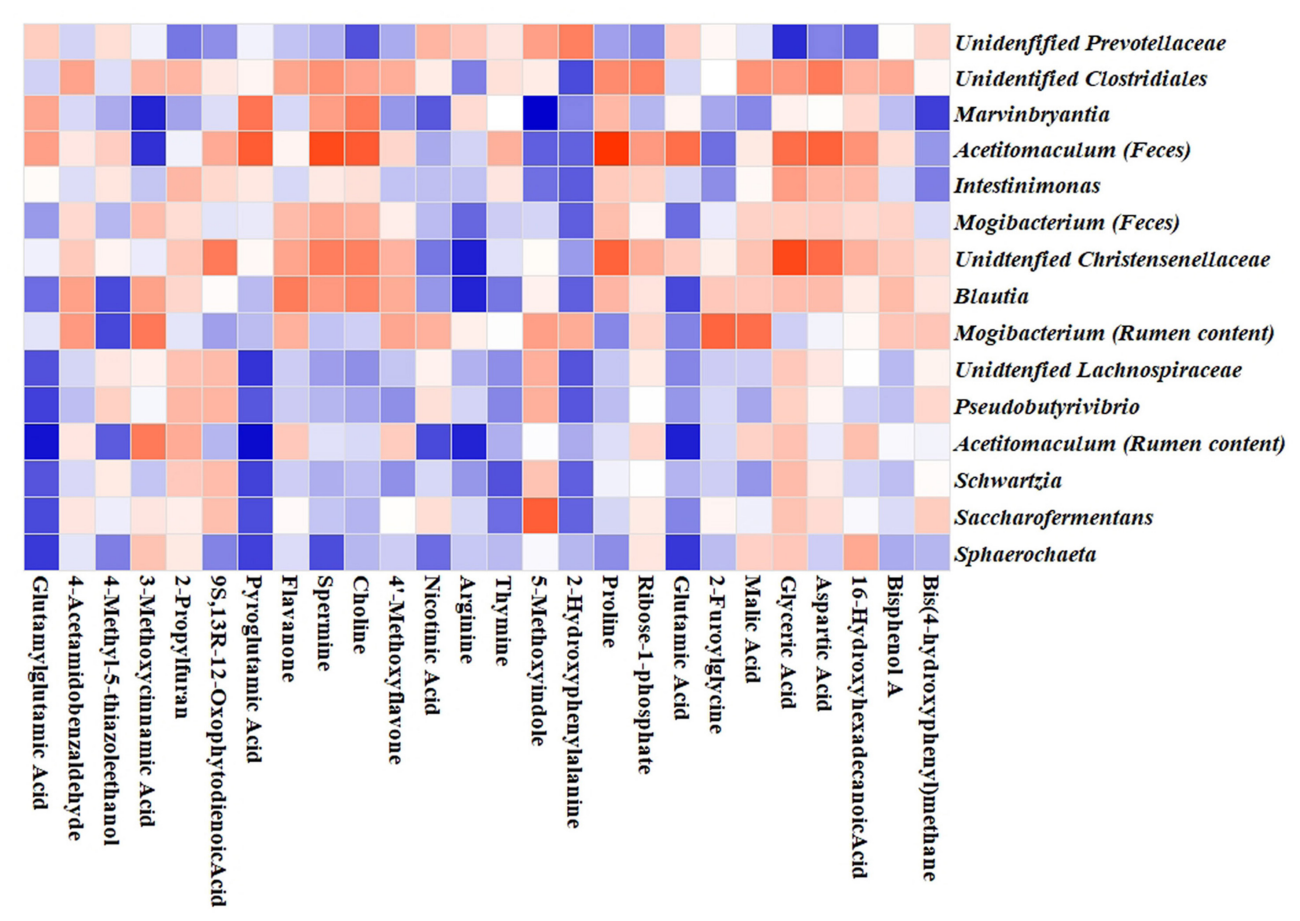
Correlation heatmap between microbiota genera and the altered metabolites. Pearson correlation between metabolites and microbiota genera in ruminal content and feces affected by NCG supplementation. Blue squares indicate negative correlations, and red squares indicate positive correlations.

Potential links among the ruminal metabolites and altered microbiota genera in ruminal content and feces were identified by Pearson correlation analysis. A clear correction with the metabolites was found for the ruminal and fecal microbiota at the genus level. As shown in Figure 4, positive correlations between metabolites and genera were indicated by the red color, and the negative correlations by blue. As shown in Figure 4, glutamylglutamic acid, pyroglutamic acid, spermine, choline, thymine, 2-hydroxyphenylalanine, and glutamic acid displayed negative corrections with the identified seven genera in the ruminal content. Three genera including *Marvinbryantia, Acetitomaculum*, and *Intestinimonas* in feces showed positive corrections with glyceric acid, spermine, choline, and aspartic acid, and negative corrections with 5-methoxyindole and 2-hydroxyphenylalanine. The association analysis between fermentation parameters and microbiota genera in ruminal content is shown in Supplementary Table S6. At the genus level, it was found that the seven genera such as *Pseudobutyrivibrio, Acetitomaculum, Schwartzia*, and *Saccharofermentans* were positively related to the changes of pH, NH_3_-N, acetate, butyrate, and the ratio of acetate/propionate, and negatively related to MCP, VFAs, and propionate.

## Discussion

Heat stress affects ruminal fermentation, metabolic profile as well as gut microbiota. As a cost-effective way, feed additive is used to ameliorate the HS in dairy cows to improve their health and production performance (28, 36). Effects of supplementation with NCG in ruminal content and feces of dairy cows under HS were studied for the first time in this study. The results found that dietary administration of NCG altered ruminal fermentation pattern, ruminal metabolic profile, and microbiota in ruminal content and feces of dairy cows exposed to HS. These findings provided evidence to illustrate the possible action mechanism of NCG in the amelioration of HS and promote the application of NCG in dairy farming. Moreover, this study integrates metabolomics and gut microbiota as an effective approach in ruminant nutritional research.

Previous studies found that HS could decrease the level of total VFAs and elevate the ratio of acetate to propionate (37, 38). In this study, the molar proportion of propionate and VFAs concentrations were increased by the NCG supplementation, while butyrate and the ratio of acetate to propionate were reduced. These results indicated that NCG could improve ruminal fermentation and have positive effects on energy production. In this study, with increasing NCG supplementation, the molar proportion of acetate and butyrate decreased. Reduced pH is conducive to the absorption of VFAs in the rumen epithelium (39). It was found NCG linearly reduced the pH in the rumen, which might be the reason for the decrease of acetate and butyrate. Propionate was increased with the addition of NCG, which was consistent with the results reported by Chacher et al. (40). As a pre-cursor of propionate, fumarate can promote propionate production in the rumen (41). In the process of gluconeogenesis, NCG is metabolized and converted to fumarate through the α-ketoglutaric acid, which may be responsible for the increase of propionate. It was noticed that ruminal pH decreased in the NCG groups, which might be resulted from the increase of the VFAs that dissociated more hydrogen ions in the rumen (42). In addition, NCG could promote nutrient digestion to increase the accumulation of organic acids in the rumen, leading to a decrease in pH (40). Oba et al. (43) reported that NCG could activate carbamyl phosphate synthetase-1 to increase the utilization of NH_3_-N in the rumen epithelial and duodenal mucosal cells, which may explain the reduced concentration of NH_3_-N in NCG groups in this study. MCP is an index to reflect microorganism growth and digestibility, and MCP synthesis is closely related to energy release. The addition of 20 g NCG/day to cows increased MCP levels in the rumen, suggesting NCG might improve the ruminal microbial community to enhance the nutrient availability for dairy cows under HS.

In our previous study, it was found that the levels of blood glucose and blood urea nitrogen were respectively increased and decreased in the dairy cows with NCG supplementation under HS (22). Propionate is the pre-cursor for glucose production. In the present study, the molar proportion of propionate was linearly increased by NCG, which might result in an increase in blood glucose. There is a linear correlation between blood urea nitrogen and rumen NH_3_-N concentration (44). It was noticed that both blood urea nitrogen and rumen NH_3_-N were reduced, which were consistent with each other and beneficial to improving the utilization rate of nitrogen.

In the present study, both Alpha diversity and PCoA indicated that NCG supplementation altered ruminal and fecal microbial community, such as the reduction of community richness and diversity. In addition, the microbial composition at the phylum and genus levels was changed by NCG. The abundance of *Proteobacteria* and *Actinobacteria* was reduced in the ruminal content in NCG groups. *Proteobacteria* have been reported to increase by HS with the presence of lipopolysaccharide in many inflammation-associated diseases (45, 46). Thus, the reduction of *Proteobacteria* caused by NCG may inhibiting inflammation to maintain the health of dairy cows. Li et al. (3) reported that phyla *Firmicutes* and *Bacteroidetes* were increased and reduced in the fecal samples of dairy cows under seasonal HS, respectively. Interestingly, a decrease in *Firmicutes* and an increase in *Bacteroidetes* were found in the feces of the dairy cows after NCG supplementation, indicating NCG reversed the dysbiosis of fecal microbiota caused by HS. At the genus level, *Mogibacterium, Sphaerochaeta, Marvinbryantia, Acetitomaculum, and Unidentified Christensenellaceae* were reduced, while *Unidentified Prevotellaceae* was enriched in NCG groups. As a pathogen, *Mogibacterium* was isolated from adult human patients with periodontal disease (47). *Marvinbryantia* plays an important role in butyrate production and energy metabolism of intestinal epithelial cells, which is associated with intestinal diseases such as colorectal cancer and ulcerative colitis (48, 49). NCG had the effect of inhibiting the level of *Mogibacterium* and *Marvinbryantia* in ruminal content and feces, indicating NCG might have the ability to protect the dairy cows from the diseases caused by potentially pathogenic bacteria.

Microbial functions of microbiota such as carbohydrate metabolism, amino acid metabolism, and energy metabolism were reduced in rumen and feces in the dairy cows or goats under HS (3). In the present study, the function of microbiota in ruminal content and feces was revealed by PICRUSt analysis, which found that the microbiota functions in NCG treatment groups differed from those in the control. Ruminal and fecal microbiota in MNCG group had an enhanced capacity to the metabolism of energy, amino acid, pyruvate, glycolysis, and carbon fixation. These results indicated that NCG could improve metabolic disorders caused by HS through the regulation of the bacterial functions in ruminal content and feces.

Metabolomic analysis in the ruminal fluid was conducted to further explore the response of rumen to NCG supplementation. The results of the score plots indicated that there were significant differences in metabolic profiles of ruminal fluid between the control and NCG groups. Twenty-six metabolites involved in arginine and proline metabolism, glutathione metabolism, citric acid cycle, glycerophospholipid metabolism, and purine metabolism were found to be associated with NCG supplementation. Moreover, the metabolomic results of ruminal fluid were partly in agreement with the findings in ruminal and fecal microbiota.

Metabolites involved in arginine metabolisms, such as arginine, aspartic acid, proline, and spermine, were affected by the NCG supplementation. Previous studies have demonstrated that NCG could increase the concentration of arginine by the activation of carbamyl phosphate synthetase-1 (19). Consistent with the previous results, the levels of arginine in the ruminal fluid were higher in NCG groups than in the control, indicating that NCG promoted the endogenous synthesis of arginine. As a toxic biogenic amine, spermine is related to subacute ruminal acidosis and can be oxidized into aldehyde and hydrogen to cause oxidative stress in ruminants (50). In the present study, NCG supplementation showed favorable inhibition of spermine concentration in the rumen, which may release less spermine into the blood to reduce oxidative stress. Proline and aspartic acid are building blocks of proteins. Chacher et al. (21) reported that dietary NCG supplementation increases aspartic acid in the plasma of the high-yield dairy cows. Inconsistent with this observation, the levels of proline and aspartic acid were reduced in the rumen of the dairy cows supplemented with NCG. The possible explanation for this discrepancy is that NCG might have different effects on the concentrations of proline and aspartic acid in plasma and ruminal content. Glutamic acid is an important nitrogen donor, it is produced from the incorporation of carbon skeleton and ammonia in the ruminal bacteria (51). In the present study, levels of glutamic acid and its derivatives such as glutamylglutamic acid and pyroglutamic acid were significantly higher in the NCG groups than in the control group, suggesting that NCG might improve the nitrogen utilization in the dairy cows under HS. As an organic acid, a high level of glyceric acid leads to general metabolic acidosis in the rumen (52). Glyceric acid was decreased in the ruminal fluid in NCG groups, which is potentially beneficial to maintaining rumen health. It was also noticed that some metabolites such as malic acid, nicotinic acid, and choline were also affected by NCG supplementation. The precise roles of these metabolites in the rumen are not clear, but the increased levels of these metabolites by NCG might be important for ruminal functions.

A great number of studies have confirmed there is a close relationship between the metabolites and gut microbiota in dairy cows (53). In the present study, correlation analysis was conducted to explore the connections between them. Our analysis revealed that a number of metabolites in ruminal fluid and feces were positively or negatively correlated with the bacterial taxa. These corrections might be important for NCG's effects on dairy cow health. *Blautia* species have been reported to be associated with glutamic acid and the transformation of host bile acids (54, 55). Consistent with the previous results, corrections among *Blautia*, choline, and glutamic acid were observed in this study, which might suggest that the reduction of *Blautia* in the feces of NCG groups had effects on choline transportation and glutamic acid production. Many ruminal metabolites were correlated with the abundance of the bacterial taxa. However, the sophisticated relationships among NCG, metabolites, and the bacterial community are poorly characterized, and further work are needed to elucidate their interactions.

## Conclusions

In the present study, an integrative approach through the combination of LC-MS-based metabolomics and 16S rRNA gene sequencing was utilized to assess the effects of NCG supplementation on microbiota and metabolites in ruminal content and feces of the dairy cows under HS. The results showed that NCG altered the ruminal fermentation pattern, including the increase of acetate, propionate, butyrate, and total VFAs and the decrease of ruminal pH and NH_3_-N. Meanwhile, NCG changed the diversity and composition of the microbiota and ameliorated the functional metabolic pathways such as amino acid metabolism, energy metabolism, and pyruvate metabolism. Metabolomic analysis revealed that 26 metabolites mainly involved in arginine metabolism, glutamate metabolism, and nitrogen metabolism were regulated by NCG. Furthermore, potential links between bacterial genera and metabolites were also revealed by correlation analysis. Combining the results of ruminal fermentation, metabolomics, and gut microbiota, the optimal dose of NCG for dairy cows under HS was 20 g/hd/day. These findings provided new insights into the effects of NCG on metabolites and microbiota in ruminal content and feces, suggesting NCG might be considered a potential dietary feed additive for the improvement of HS in dairy cows. Further research are required to investigate the underlying interactions among NCG, metabolites, and microbiota.

## Data Availability Statement

The datasets presented in this study can be found in online repositories. The names of the repository/repositories and accession number(s) can be found below: https://www.ncbi.nlm.nih.gov/, PRJNA811687; https://www.ncbi.nlm.nih.gov/, PRJNA817393.

## Ethics Statement

The animal study was reviewed and approved by the Institutional Animal Care and Use Committee of Hebei Agricultural University and carried out in accordance with the Guidelines of the Care and Use of Laboratory Animals of China.

## Author Contributions

YG and JL: conceptualization, methodology, supervision, and funding acquisition. YL, LR, NM, MW, and LH: investigation and data curation. YC, QL, and YS: resources. LH, NM, and YL: writing and editing. All authors read and approved the final manuscript.

## Funding

This research was financially supported by the earmarked fund for China Agriculture Research System (CARS-36, Beijing, China), Hebei Dairy Cattle Innovation Team of Modern Agro-industry Technology Research System (HBCT2018120203, Shijiazhuang, China), Key Research and Development Project of Hebei (20326606D, Shijiazhuang, China), and Precision Animal Husbandry Discipline Group Construction Project of Hebei Agricultural University (2020, Hebei, China).

## Conflict of Interest

The authors declare that the research was conducted in the absence of any commercial or financial relationships that could be construed as a potential conflict of interest.

## Publisher's Note

All claims expressed in this article are solely those of the authors and do not necessarily represent those of their affiliated organizations, or those of the publisher, the editors and the reviewers. Any product that may be evaluated in this article, or claim that may be made by its manufacturer, is not guaranteed or endorsed by the publisher.
