# Supplementary material for: Microbiome-Metabolome Responses in Ruminal Content and Feces of Lactating Dairy Cows With N-Carbamylglutamate Supplementation Under Heat Stress
Source: Front Vet Sci. 2022 Jun 23;9:902001. doi: 10.3389/fvets.2022.902001 (PMC9260145; doi:10.3389/fvets.2022.902001)
Supplement: Supplementary file 1 [file Data_Sheet_1.docx]

***Supplementary material S1***

**Liquid chromatography conditions**

Chromatographic separation of plasma was performed on Vanquish ultra high-performance liquid chromatograph (UHPLC) system. Elution was carried out on an Accucore HILIC column (2.1×150 mm, 2.6 μm) maintained at 40°C.

For the positive ion mode, the mobile phase was consisted of A (0.1% formic acid, 95% acetonitrile and 10 mM ammonium acetate) and B (0.1% formic acid, 50% acetonitrile and 10 mM ammonium acetate); the negative ion mode was consisted of A (95% acetonitrile and 10 mM ammonium acetate) and B (50% acetonitrile and 10 mM ammonium acetate). The flow rate was 0.3 mL/min following linear gradient elution program: 0-1 min, 98% A; 1-17 min, 98-50% A; 17-17.5 min, 50% A; 17.5-18 min, 50-98% A; 18-20 min, 98% A.

**Mass spectrometry conditions**

Q ExactiveTM HF-X mass spectrometer (Thermo Fisher, Waltham, MA, USA) operating in positive (ESI+) and negative ion modes (ESI-) was used in this study. The main MS parameters were set as follows: spray voltage, 3.2 KV; sheath gas flow rate, 35 arb; aux gas flow rate, 10 arb; scan range, m/z of 100-1500; capillary temperature, 320°C. MS/MS data was acquired in data dependent acquisition mode.

**Metabonomic data processing**

The raw MS data was converted and processed by Compound Discovery 2.0 (Thermo Fisher, Waltham, MA, USA). Nonlinear alignment, automatic integration and extraction of the peak intensities of the MS data was performed to generate a dataset. Then, SIMCA-P (Umetrics AB, Umea, Sweden) was used for multivariate statistical analysis of the obtained dataset including principal component analysis (PCA) and partial least squares discriminant analysis (PLS-DA). Cumulative R2 and Q2 of the PCA and PLS-DA models were used to describe the fitness and predictive capability of the model. Based on the PLS-DA model, the variable importance in projection (VIP) score of the metabolites was constructed. Meanwhile, the P-value of the metabolite was also analyzed by Student’s t-test. With VIP > 1 and P < 0.05, the metabolites were considered to be statistically significant different.

***Supplementary material S2***

**Table S1** Composition and nutrient levels of the basal diet.

| Item | Diet |
| --- | --- |
| Ingredient, % of DM  Whole corn silage | 25.00 |
| Oat hay | 2.00 |
| Alfalfa hay | 15.00 |
| Cracked Corn | 13.58 |
| Whole cotton seed | 5.93 |
| Steam flaked corn | 9.18 |
| Wheat bran | 1.39 |
| Soybean meal | 9.21 |
| Dried beet pellet | 5.38 |
| Rapeseed meal | 1.53 |
| Corn DDGS^1^ | 1.65 |
| Premix^2^ | 1.00 |
| Extruded soybean | 4.10 |
| Molasses | 1.00 |
| Fat powder | 2.26 |
| Limestone | 0.29 |
| Calcium phosphate | 0.56 |
| MgO | 0.14 |
| NaHCO_3_ | 0.59 |
| NaCl | 0.20 |
| Mold remover | 0.01 |
| Total | 100.00 |
| Chemical composition, DM basis |  |
| NE_L_, Mcal/kg ^3^ | 1.64 |
| Crude protein, % | 17.02 |
| Ether extract, % | 5.13 |
| Neutral detergent fiber, % | 34.48 |
| Acid detergent fiber, % | 20.44 |
| Calcium, % | 0.68 |
| Phosphorus, % | 0.37 |

^1^DDGS = dried distillers grains with solubles.

^2^Premix contained (per kg of DM): 800,000 IU of vitamin A, 180,000 IU of vitamin D3, 7,000 mg of vitamin E, 45 mg of biotin, 300 mg of β-carotene, 600 mg of Cu, 1,000 mg of Fe, 2,200 mg of Zn, 1,800 mg of Mn, 20 mg of Co, 30 mg of Se and 39 mg of I.

^3^Data were calculated according to NRC models (NRC, 2001).


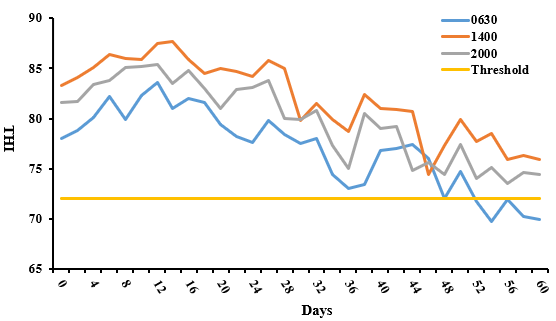
**Figure S1** THI curve of cowsheds during the whole experiment.

THI: temperature-humidity index. The THI values in the morning (0630), midday (1400) and night (2000) were recorded in the experiment and all the THI values were greater than 72 (threshold), indicating the dairy cows were exposed to the heat stress.

**Table S2** Summary of the sequencing data of rumen content and feces.

| Sample ID | Raw PE reads | Raw Tags | Clean Tags | Effective Tags | OTU numbers |
| --- | --- | --- | --- | --- | --- |
| *Rumen content* | | | | | |
| Control-1 | 89,106 | 85,943 | 83,372 | 62,078 | 2,329 |
| Control-2 | 91,194 | 88,372 | 85,981 | 64,973 | 2,388 |
| Control-3 | 98,560 | 94,286 | 91,430 | 75,355 | 2,445 |
| Control-4 | 81,408 | 78,513 | 76,381 | 58,809 | 2,222 |
| LNCG-1 | 98,550 | 95,547 | 93,149 | 72,801 | 2,034 |
| LNCG-2 | 89,706 | 85,009 | 82,544 | 64,882 | 2,375 |
| LNCG-3 | 91,157 | 88,076 | 85,687 | 70,162 | 2,197 |
| LNCG-4 | 96,576 | 93,874 | 91,449 | 73,374 | 2,308 |
| LNCG-5 | 86,670 | 84,596 | 82,053 | 73,313 | 2,079 |
| MNCG-1 | 81,334 | 79,232 | 77,111 | 58,882 | 1,650 |
| MNCG-2 | 94,122 | 90,445 | 87,836 | 74,421 | 2,059 |
| MNCG-3 | 84,236 | 81,715 | 79,324 | 64,607 | 2,091 |
| MNCG-4 | 97,821 | 94,962 | 92,561 | 66,340 | 2,120 |
| MNCG-5 | 96,908 | 94,383 | 92,184 | 70,589 | 2,246 |
| HNCG-1 | 94,917 | 92,353 | 90,063 | 69,724 | 2,124 |
| HNCG-2 | 99,645 | 96,632 | 94,298 | 72,399 | 2,166 |
| HNCG-3 | 85,538 | 83,380 | 81,469 | 61,393 | 1,809 |
| HNCG-4 | 97,884 | 95,192 | 92,765 | 76,346 | 2,307 |
| HNCG-5 | 92,603 | 89,067 | 86,802 | 70,965 | 2,080 |
| *Feces* | | | | | |
| Control-1 | 90,916 | 87,680 | 84,864 | 73,261 | 2,431 |
| Control-2 | 92,354 | 89,259 | 86,470 | 76,178 | 2,539 |
| Control-3 | 85,255 | 82,433 | 79,789 | 70,116 | 2,307 |
| Control-4 | 93,970 | 90,644 | 87,632 | 77,257 | 2,378 |
| Control-5 | 82,436 | 79,734 | 77,414 | 65,881 | 2,281 |
| LNCG-1 | 91,575 | 88,504 | 85,807 | 74,824 | 2,407 |
| LNCG-2 | 85,558 | 82,815 | 80,354 | 68,457 | 2,435 |
| LNCG-3 | 82,545 | 80,275 | 77,716 | 70,230 | 2,304 |
| LNCG-4 | 88,501 | 86,147 | 83,630 | 73,883 | 1,986 |
| LNCG-5 | 83,825 | 81,638 | 79,236 | 66,386 | 1,987 |
| MNCG-1 | 92,201 | 89,731 | 86,811 | 80,135 | 2,131 |
| MNCG-2 | 92,304 | 89,178 | 86,044 | 77,922 | 2,383 |
| MNCG-3 | 97,967 | 94,852 | 92,268 | 81,964 | 2,227 |
| MNCG-4 | 98,471 | 95,811 | 93,286 | 85,154 | 2,330 |
| MNCG-5 | 82,563 | 80,157 | 77,939 | 70,727 | 2,148 |
| HNCG-1 | 92,077 | 89,055 | 85,940 | 77,417 | 2,285 |
| HNCG-2 | 84,809 | 82,342 | 80,160 | 70,898 | 2,073 |
| HNCG-3 | 95,943 | 93,109 | 90,723 | 82,093 | 2,379 |
| HNCG-4 | 90,898 | 88,336 | 86,344 | 76,786 | 2,326 |
| HNCG-5 | 82,013 | 79,871 | 77,849 | 71,222 | 2,111 |

NCG: N-carbamylglutamate, Control: without NCG supplementation, LNCG: NCG at 15 g/d per cow, MNCG: NCG at 20 g/d per cow, HNCG: NCG at 25 g/d per cow.

| 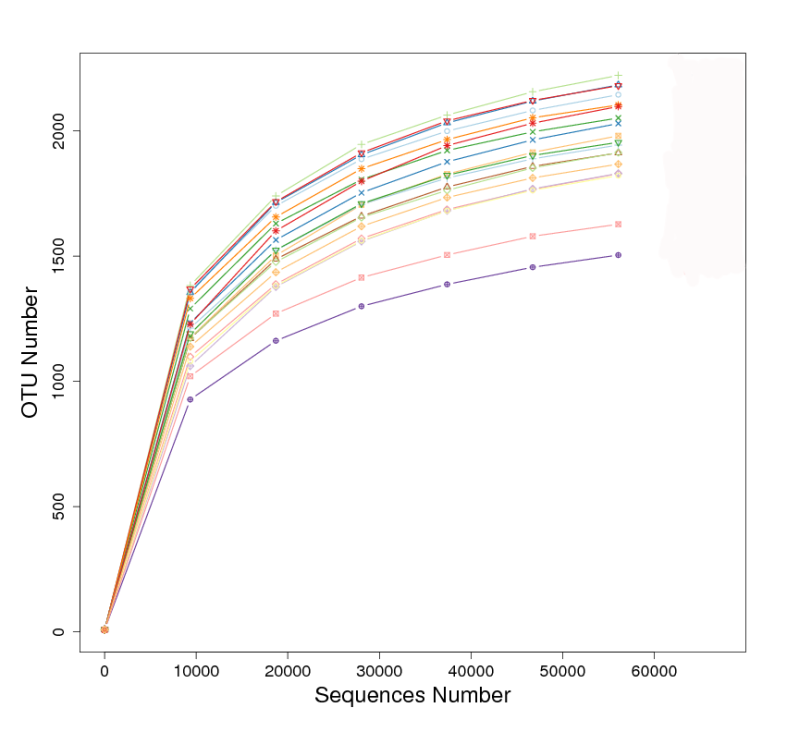  **a** | 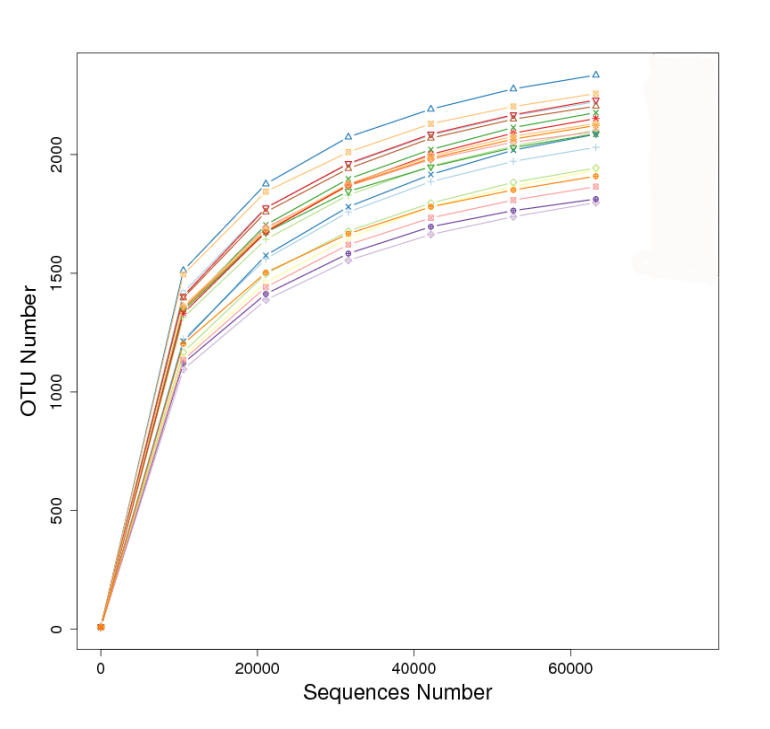  **b** |
| --- | --- |

**Figure S2** Rarefaction curve of the samples from rumen content and feces.

a: rarefaction curve of rumen content samples; b: rarefaction curve of fecal samples. OUT numbers gradually increased with the increase of sequence number, and the curve became gentle, indicating the obtained sequence could reflect the abundance and diversity of the microbiota.

**Table S3** Relative abundance of ruminal and fecal microbiota at phylum level in different group.

| Taxon | Groups | | | | SEM | *P*-value | | | |
| --- | --- | --- | --- | --- | --- | --- | --- | --- | --- |
|  | Control | LNCG | MNCG | HNCG |  | *P*-NCG | Linear | Quadratic | Cubic |
| Rumen content |  |  |  |  |  |  |  |  |  |
| *Firmicutes* | 48.34 | 46.51 | 53.58 | 50.27 | 4.69 | 0.73 | 0.63 | 0.80 | 0.34 |
| *Bacteroidetes* | 39.98 | 40.71 | 38.97 | 36.52 | 1.87 | 0.42 | 0.30 | 0.22 | 0.96 |
| *Proteobacteria* | 4.24 | 2.46 | 1.87 | 2.13 | 0.673 | 0.14 | 0.03 | 0.46 | 0.70 |
| *Spirochaetes* | 1.80 | 2.14 | 1.20 | 1.18 | 0.371 | 0.22 | 0.22 | 0.22 | 0.23 |
| *Tenericutes* | 1.82 | 1.52 | 1.51 | 1.43 | 0.252 | 0.75 | 0.29 | 0.87 | 0.89 |
| *Verrucomicrobia* | 0.47 | 0.53 | 0.30 | 0.61 | 0.229 | 0.79 | 0.90 | 0.72 | 0.36 |
| *Fibrobacteres* | 0.29 | 0.40 | 0.11 | 0.13 | 0.143 | 0.43 | 0.39 | 0.37 | 0.30 |
| *Actinobacteria* | 0.39 | 0.26 | 0.12 | 0.1 | 0.079 | 0.08 | 0.01 | 0.81 | 0.54 |
| *Melainabacteria* | 0.34 | 0.21 | 0.10 | 0.13 | 0.069 | 0.12 | 0.03 | 0.82 | 0.45 |
| *Euryarchaeota* | 0.16 | 0.19 | 0.33 | 0.21 | 0.074 | 0.40 | 0.36 | 0.61 | 0.17 |
| *Others* | 2.19 | 1.70 | 1.89 | 1.75 | 0.179 | 0.29 | 0.12 | 0.42 | 0.36 |
| Feces |  |  |  |  |  |  |  |  |  |
| *Bacteroidetes* | 47.73 | 51.02 | 61.04 | 56.87 | 3.81 | 0.10 | 0.04 | 0.98 | 0.14 |
| *Firmicutes* | 43.68 | 41.61 | 28.65 | 35.00 | 3.71 | 0.04 | 0.03 | 0.95 | 0.05 |
| *Proteobacteria* | 2.02 | 1.72 | 5.12 | 2.01 | 1.13 | 0.15 | 0.48 | 0.64 | 0.04 |
| *Spirochaetes* | 2.11 | 1.44 | 1.72 | 1.66 | 0.400 | 0.70 | 0.41 | 0.49 | 0.65 |
| *Tenericutes* | 2.27 | 1.55 | 1.77 | 2.19 | 0.260 | 0.20 | 0.51 | 0.04 | 0.97 |
| *Euryarchaeota* | 0.39 | 0.81 | 0.26 | 0.47 | 0.194 | 0.26 | 0.98 | 0.29 | 0.09 |
| *Kiritimatiellaeota* | 0.16 | 0.14 | 0.22 | 0.10 | 0.069 | 0.70 | 0.84 | 0.60 | 0.30 |
| *Fibrobacteres* | 0.11 | 0.18 | 0.15 | 0.20 | 0.050 | 0.68 | 0.29 | 0.99 | 0.55 |
| *Oxyphotobacteria* | 0.06 | 0.10 | 0.04 | 0.13 | 0.054 | 0.61 | 0.55 | 0.76 | 0.25 |
| *unidentified_Bacteria* | 0.28 | 0.28 | 0.18 | 0.28 | 0.043 | 0.30 | 0.58 | 0.54 | 0.09 |
| *Others* | 1.19 | 1.14 | 0.85 | 1.07 | 0.168 | 0.51 | 0.38 | 0.78 | 0.24 |

NCG: N-carbamylglutamate, Control: without NCG supplementation, LNCG: NCG at 15 g/d per cow, MNCG: NCG at 20 g/d per cow, HNCG: NCG at 25 g/d per cow. Relative abundance was represented by the percent of total sequences.

| **a**  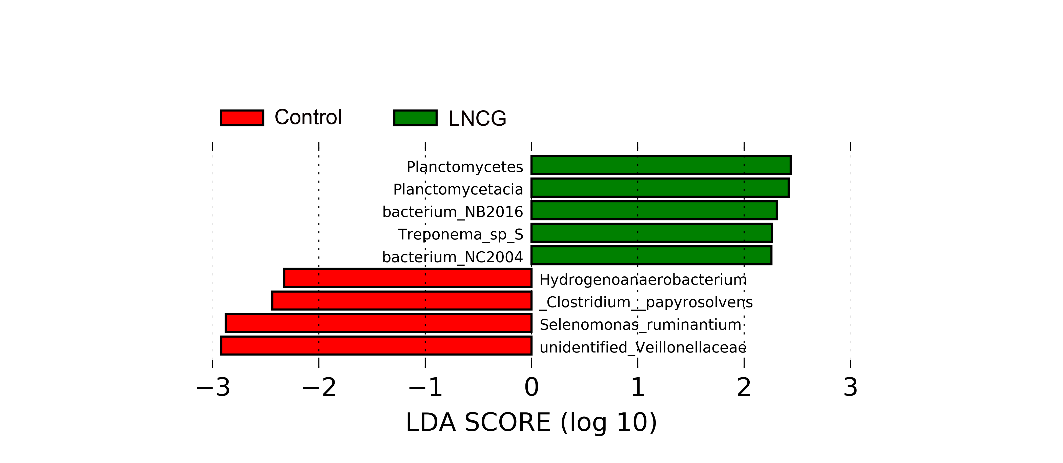 |
| --- |
| 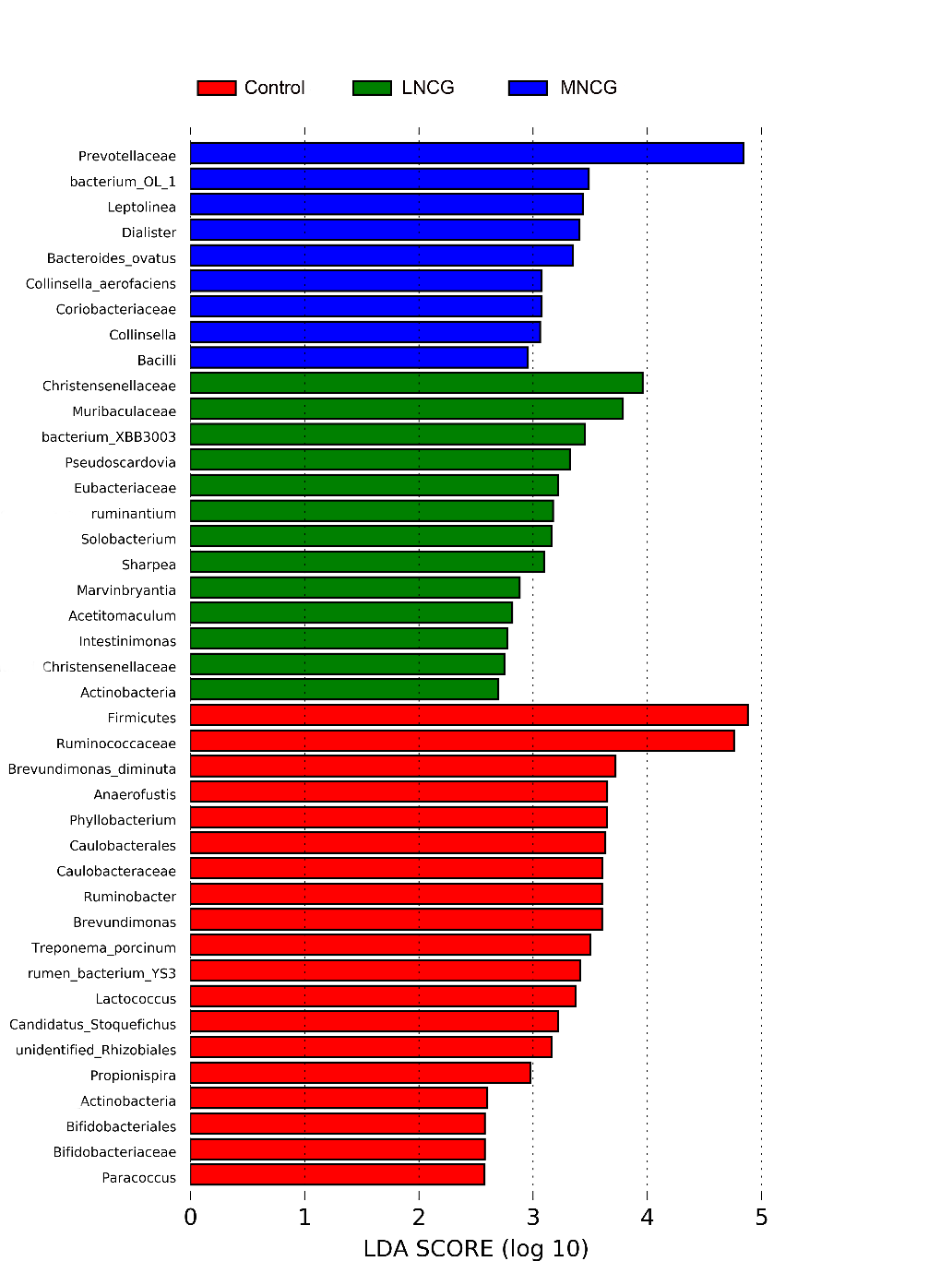  **b** |

**Figure S3** Bacterial taxa significantly differentiated in rumen content and feces between the control and NCG groups identified by LEfSe.

NCG: N-carbamylglutamate, Control: without NCG supplementation, LNCG: NCG at 15 g/d per cow, MNCG: NCG at 20 g/d per cow, HNCG: NCG at 25 g/d per cow. a: different taxa in the rumen content; b: different taxa in the feces. Only taxa meeting an LDA significant threshold of 2 are shown.

**Table S4** Relative abundance of ruminal and fecal microbiota at genus level in different group.

| Taxon | Groups | | | | SEM | *P*-value | | | |
| --- | --- | --- | --- | --- | --- | --- | --- | --- | --- |
|  | Control | LNCG | MNCG | HNCG |  | *P*-NCG | Linear | Quadratic | Cubic |
| Rumen content |  |  |  |  |  |  |  |  |  |
| *Mogibacterium* | 0.08 | 0.04 | 0.06 | 0.05 | 0.006 | 0.01 | ＜0.01 | 0.11 | 0.06 |
| *Unidentified Lachnospiraceae* | 1.37 | 1.24 | 0.86 | 0.97 | 0.188 | 0.25 | 0.10 | 0.88 | 0.27 |
| *Pseudobutyrivibrio* | 0.15 | 0.13 | 0.08 | 0.10 | 0.031 | 0.37 | 0.15 | 0.91 | 0.32 |
| *Acetitomaculum* | 0.10 | 0.08 | 0.06 | 0.07 | 0.013 | 0.46 | 0.18 | 0.78 | 0.41 |
| *Schwartzia* | 0.03 | 0.03 | 0.01 | 0.02 | 0.008 | 0.27 | 0.22 | 0.71 | 0.14 |
| *Saccharofermentans* | 0.36 | 0.27 | 0.21 | 0.21 | 0.048 | 0.15 | 0.03 | 0.82 | 0.56 |
| *Sphaerochaeta* | 0.04 | 0.02 | 0.02 | 0.01 | 0.005 | 0.02 | ＜0.01 | 0.93 | 0.85 |
| Feces |  |  |  |  |  |  |  |  |  |
| *Unidentified Prevotellaceae* | 10.07 | 13.70 | 27.25 | 23.15 | 2.92 | <0.01 | <0.01 | 0.67 | 0.03 |
| *Unidentified Clostridiales* | 0.19 | 0.16 | 0.11 | 0.17 | 0.028 | 0.33 | 0.29 | 0.36 | 0.21 |
| *Marvinbryantia* | 0.12 | 0.13 | 0.05 | 0.08 | 0.019 | 0.03 | 0.05 | 0.33 | 0.02 |
| *Acetitomaculum* | 0.14 | 0.18 | 0.08 | 0.11 | 0.020 | 0.01 | 0.12 | 0.09 | 0.01 |
| *Intestinimonas* | 0.07 | 0.08 | 0.03 | 0.06 | 0.013 | 0.06 | 0.33 | 0.61 | 0.01 |
| *Mogibacterium* | 0.07 | 0.07 | 0.04 | 0.04 | 0.011 | 0.16 | 0.07 | 0.58 | 0.19 |
| *Unidentified Christensenellaceae* | 0.09 | 0.09 | 0.06 | 0.06 | 0.007 | 0.01 | 0.02 | 0.14 | 0.01 |
| *Blautia* | 0.06 | 0.05 | 0.03 | 0.04 | 0.013 | 0.35 | 0.15 | 0.67 | 0.33 |

NCG: N-carbamylglutamate, Control: without NCG supplementation, LNCG: NCG at 15 g/d per cow, MNCG: NCG at 20 g/d per cow, HNCG: NCG at 25 g/d per cow. Relative abundance was represented by the percent of total sequences.

**
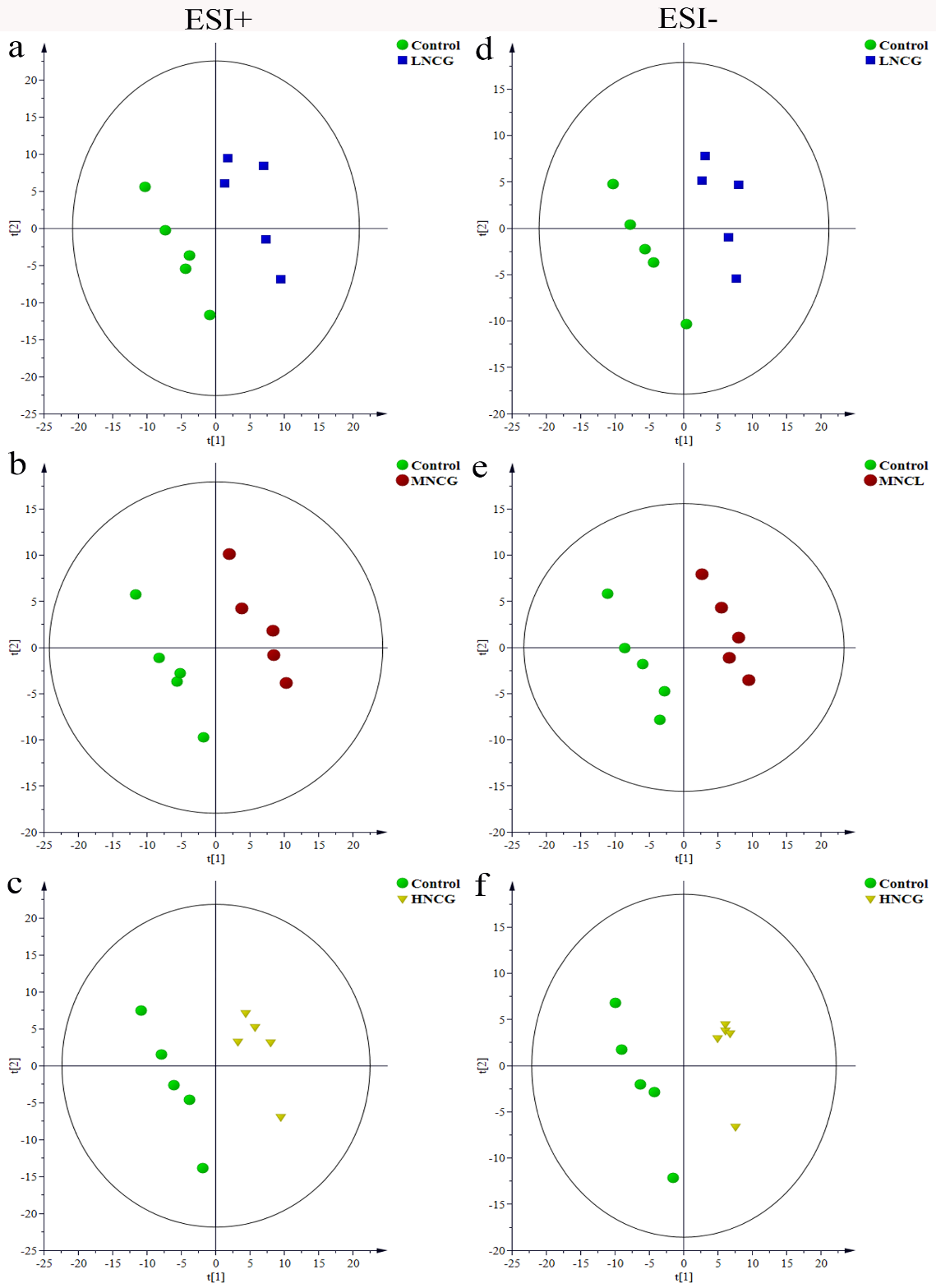
Figure S4** PLS-DA score plots of rumen fluid samples from different group.

NCG: N-carbamylglutamate, Control: without NCG supplementation, LNCG: NCG at 15 g/d per cow, MNCG: NCG at 20 g/d per cow, HNCG: NCG at 25 g/d per cow. PLS-DA score plots derived from UPLC-Q-TOF/MS in positive (ESI+, a-c) and negative (ESI-, d-f) ionization modes. Samples in control and NCG groups were clearly separated in the score plots.

Table S5 Identification of the different metabolites in the rumen content related to NCG supplementation of the dairy cows under heat stress

| Metabolites | IM | Formula | MW | RT | Fold change | | | Metabolic pathway |
| --- | --- | --- | --- | --- | --- | --- | --- | --- |
|  |  |  |  |  | LNCG/C | MNCG/C | HNCG/C |  |
| Glutamylglutamic acid | ESI+ | C_10_H_16_N_2_O_7_ | 276.0949 | 1.15 | 1.94^*^ | 1.70 | 1.26 | — |
| 4-Acetamidobenzaldehyde | ESI+ | C_9_H_9_NO_2_ | 163.0631 | 8.51 | 0.59^*^ | 0.64 | 0.68 | — |
| 4-Methyl-5-thiazoleethanol | ESI+ | C_6_H_9_NOS | 143.0403 | 3.38 | 1.54^*^ | 1.20 | 0.91 | Thiamine metabolism |
| 3-Methoxycinnamic acid | ESI+ | C_10_H_10_O_3_ | 178.0627 | 8.02 | 0.56^*^ | 0.75^*^ | 0.75^*^ | — |
| 2-Propylfuran | ESI+ | C_7_H_10_O | 110.0730 | 8.90 | 0.75^*^ | 0.59^**^ | 0.73^**^ | — |
| 9S,13R-12-Oxophytodienoic acid | ESI+ | C_18_H_28_O_3_ | 292.2029 | 14.08 | 0.90 | 0.46^*^ | 0.65 | — |
| Pyroglutamic acid | ESI+ | C_5_H_7_NO_3_ | 129.0425 | 1.53 | 3.20^**^ | 3.03^**^ | 3.87^*^ | Glutathione metabolism |
| Flavanone | ESI+ | C_15_H_12_O_2_ | 224.0830 | 9.42 | 0.63^*^ | 0.59^*^ | 0.58^*^ | — |
| Spermine | ESI+ | C_10_H_26_N_4_ | 202.2153 | 0.91 | 1.01 | 0.57^**^ | 0.61^**^ | Arginine and proline metabolism |
| Choline | ESI+ | C_5_H_13_NO | 103.0996 | 1.03 | 0.83 | 0.50^*^ | 0.58^*^ | Glycerophospholipid metabolism |
| 4'-Methoxyflavone | ESI+ | C_16_H_12_O_3_ | 252.0779 | 9.76 | 0.71^*^ | 0.75 | 0.66^**^ | — |
| Nicotinic acid | ESI+ | C_6_H_5_NO_2_ | 123.0319 | 1.33 | 0.74 | 0.75 | 0.62^*^ | Nicotinate and nicotinamide metabolism |
| Arginine | ESI+ | C_6_H_14_N_4_O_2_ | 174.1114 | 1.02 | 1.21 | 1.48 | 1.63^*^ | Arginine and proline metabolism |
| Thymine | ESI+ | C_5_H_6_N_2_O_2_ | 126.0428 | 1.82 | 0.92 | 0.82 | 0.59 | Pyrimidine metabolism |
| 5-Methoxyindole | ESI+ | C_9_H_9_NO | 147.0682 | 9.54 | 0.45^*^ | 0.58 | 0.51^*^ | — |
| 2-Hydroxyphenylalanine | ESI+ | C_9_H_11_NO_3_ | 181.0737 | 1.45 | 1.04 | 1.37 | 0.54 | — |
| Proline | ESI+ | C_5_H_9_NO_2_ | 115.0632 | 1.08 | 1.07 | 0.71 | 0.66 | Arginine and proline metabolism |
| Ribose-1-phosphate | ESI- | C_5_H_11_O_8_P | 230.0194 | 0.98 | 0.52^*^ | 0.50^*^ | 0.60 | Purine metabolism |
| Glutamic acid | ESI- | C_5_H_9_NO_4_ | 147.0535 | 0.98 | 9.91^*^ | 9.03^**^ | 8.38^**^ | Arginine biosynthesis |
| 2-Furoylglycine | ESI- | C_7_H_7_NO_4_ | 169.0379 | 0.99 | 0.48^*^ | 0.44^*^ | 0.43^**^ | — |
| Malic acid | ESI- | C_4_H_6_O_5_ | 134.0218 | 0.99 | 0.41^*^ | 0.41^*^ | 0.34^*^ | Citric Acid Cycle |
| Glyceric acid | ESI- | C_3_H_6_O_4_ | 106.0269 | 1.00 | 0.72 | 0.35^**^ | 0.49^**^ | Glycerolipid metabolism |
| Aspartic acid | ESI- | C_4_H_7_NO_4_ | 133.0378 | 0.98 | 0.79 | 0.61^**^ | 0.63^*^ | Arginine biosynthesis |
| 16-Hydroxyhexadecanoic acid | ESI- | C_16_H_32_O_3_ | 272.2353 | 14.04 | 0.80 | 0.58^*^ | 0.61 | — |
| Bisphenol A | ESI- | C_15_H_16_O_2_ | 228.1154 | 8.94 | 0.76 | 0.71 | 0.61^*^ | Bisphenol degradation |
| Bis(4-hydroxyphenyl)methane | ESI- | C_13_H_12_O_2_ | 200.0841 | 8.12 | 0.73 | 0.71 | 0.64^*^ | — |

NCG: N-carbamylglutamate, C: control group without NCG supplementation, LNCG: NCG at 15 g/d per cow, MNCG: NCG at 20 g/d per cow, HNCG: NCG at 25 g/d per cow IM: ion mode, MW: molecular weight, RT: retention time. Compared with the control, ^*^*P* < 0.05, ^**^*P* < 0.01.

Table S6 Pearson correlation analysis between fermentation parameters and microbiota genera in ruminal content under dietary supplementation of NCG.

| Items | pH | NH3-N | MCP | VFAs | Acetate | Propionate | Butyrate | Acetate/Propionate |
| --- | --- | --- | --- | --- | --- | --- | --- | --- |
| *Mogibacterium* | 0.748 | 0.519 | -0.204 | -0.463 | 0.815 | -0.701 | 0.405 | 0.723 |
| *Unidentified Lachnospiraceae* | 0.874 | 0.959* | -0.742 | -0.724 | 0.785 | -0.783 | 0.981* | 0.753 |
| *Pseudobutyrivibrio* | 0.878 | 0.975* | -0.795 | -0.780 | 0.808 | -0.822 | 0.994** | 0.794 |
| *Acetitomaculum* | 0.954* | 0.999** | -0.798 | -0.845 | 0.922 | -0.922 | 0.988* | 0.904 |
| *Schwartzia* | 0.690 | 0.879 | -0.827 | -0.729 | 0.612 | -0.680 | 0.933 | 0.645 |
| *Saccharofermentans* | 0.987* | 0.957* | -0.630 | -0.714 | 0.926 | -0.866 | 0.934 | 0.849 |
| *Sphaerochaeta* | 0.945 | 0.785 | -0.327 | -0.490 | 0.881 | -0.742 | 0.729 | 0.736 |

MCP: microbial protein, VFAs: total volatile fatty acids. *p < 0.05 and **p < 0.01 according to Pearson correlation.
